# Development of Culturally Adapted Media to Increase Community Awareness of Childhood Cancer in Tanzania

**DOI:** 10.1200/GO.22.49000

**Published:** 2022-05-05

**Authors:** Dalia Abualhaj, Erica Sanga, Shabani Rajabu, Francis Karia, Christina Makarushka, Kathryn Pollak, Laura Fish, Kristin Schroeder

**Affiliations:** ^1^ Duke Global Health Institute, Duke University, Durham, NC; ^2^ National Institute for Medical Research, Lake Victoria, Tanzania; ^3^ Bugando Medical Centre, Mwanza, Tanzania; ^4^ Duke Office of Clinical Research, Duke University, Durham, NC; ^5^ Department of Family Medicine and Community Health, Duke University, Durham, NC; ^6^ Department of Population Health Sciences, Duke University, Durham, NC; ^7^ Department of Pediatric Oncology, Duke University Hospital, Department of Global Health, Duke University, Durham, NC

## Abstract

There is low childhood cancer awareness in many low and middle income countries (LMICs), contributing to delayed hospital presentation, and treatment abandonment. In Tanzania, only 20% of the estimated 3,500 new cases per year present for treatment. While childhood cancer awareness media exist, they are not adapted for use in many LMIC communities, including Tanzania.

**METHODS:**

To reach consensus on childhood cancer education content and education strategy selection, a Delphi process was completed with an expert advisory panel representing three Tanzanian referral hospitals. Focus groups discussions (FGD) were completed with rural and urban communities from each of the Mwanza, Dar Es Salaam and Kilimanjaro regions. Thematic analysis was guided by content consensus to identify community knowledge gaps. Sample education materials were reviewed to identify preferred delivery format.

**RESULTS:**

A total of 14 and 72 participants formed the expert panel and community FGD respectively. Education strategies with the highest feasibility and impact included community health worker led education, a song, and social media messaging. The following key knowledge content areas were identified to have the lowest community awareness. (1) The cause of most childhood cancer is unknown: 83% felt untreated illnesses, cooking oil, witchcraft, immune system deficiency, and the environment were causes of childhood cancer; (2) Symptom recognition and hospital based studies can improve early detection: 66% reported that ill children see a traditional healer or pharmacist and only go a hospital if febrile or if symptoms do not improve; (3) Most childhood cancer can be cured with early detection: 50% felt that there is no cure.

**CONCLUSION:**

This study identified key childhood cancer knowledge gaps within the Tanzanian community and culturally adapted strategies to disseminate education media. Additional studies are needed to evaluate the impact of culturally adapted media on community awareness and early hospital presentation in the region.

